# Vitamin D status in children^⋆^

**DOI:** 10.1016/j.jped.2024.04.001

**Published:** 2024-04-08

**Authors:** Roger Bouillon, Leen Antonio, Nick Narinx

**Affiliations:** Clinical and Experimental Endocrinology, Department of Chronic Diseases and Metabolism, KU Leuven, Leuven, Belgium

Vitamin D was identified, about a century ago, as the agent able to cure nutritional rickets that was endemic in many regions around the world. Cod liver oil or pharmaceutical vitamin D(2) were rapidly introduced as curative agents for the prevention of rickets in many countries as a “national” health policy so that nutritional rickets were largely eradicated in countries and regions with such a strategy. Rickets remains a problem in regions or subgroups that for whatever reason do not implement such a strategy.^1^, ^2^, ^3^ The authors now know that vitamin D is an inactive precursor for 25-hydroxyvitamin D (25(OH)D), used as the best indicator for vitamin D status, and 1,25-dihydroxyvitamin D (1,25(OH)_2_D), the active hormone acting on the vitamin D receptor (VDR). The authors now also realize that activation of the VDR regulates a very large number of genes as about 3 % of the mouse or humans, and even about 10 % of the young zebrafish, genome is directly or indirectly under the control of 1,25(OH)_2_D. These genes are usually clusters of genes involved in a wide variety of functions, such as cell replication, immune function, and cell differentiation.^4^ These genetic data in combination with a wealth of studies on cellular, tissue, and whole-body functions lead to the hypothesis that vitamin D might well be implicated in many physiologic functions, and that vitamin D deficiency may contribute to a very large number of major health problems such as cancer, immune, metabolic or vascular diseases.^4^

Evidently, it became important to evaluate the vitamin D status of subjects around the world and to define a threshold below (or above) which the vitamin D status would generate negative health effects. In addition, many observational studies needed to be complemented by large randomized controlled trials to define which diseases may be avoided by vitamin D supplements given to populations with different risk factors and vitamin D status. Hundreds of publications have generated a wealth of data on the vitamin D status of people living around the world. Most of our vitamin D originates from its synthesis in the skin during exposure to ultraviolet (UV) B sunlight. One might thus expect that people living in countries with plenty of sunshine would have much higher serum 25(OH)D concentrations and a low rate of real deficiency. In reality, the situation is more complex. In Europe, vitamin D status is even lower in Mediterranean countries than in the Scandinavian population and the risk of vitamin D deficiency is much greater in North Africa and Middle East or Gulf countries than in many other countries.^5^ Differences in lifestyle, including clothing habits and avoidance of direct sun exposure, are the main driving factors for these differences. Based on its location and plenty of sunshine, Africa was supposed to be virtually free of vitamin D deficiency. However, an overview of all publications dealing with 25(OH)D concentrations in African countries, revealed surprising results with a higher than predicted rate of deficiencies in South Africa and especially North African countries.^6^ When compared with other continents or large regions of the world, the vitamin D status of the African population was not better. Very few studies found mean serum 25(OH)D concentrations that were supposed to be historic levels of early humans as identified by mean serum 25OHD levels of about 45 ng/mL in people with a traditional lifestyle such as the Masai and similar tribes.^7^ In the overviews following these publications, data from South or Latin America were largely missing.

In the present issue of this journal, Radonsky et al. present data obtained from 413,976 children, aged 0 – 18 years, from different regions of Brazil and thereby generate data to fill one of the major missing gaps to complete a worldwide overview of vitamin D status.^8^ The children were living in 6 major urban areas and children from rural areas were not included. The mean serum 25(OH)D was 29 ± 9 ng/mL, 12.0 % of the children were vitamin D deficient (serum 25(OH)D below 20 ng/mL) and 0.8 % had severe vitamin D deficiency (serum 25(OH)D below 12 ng/mL). They found an expected 20.0 % variation due to season (obviously according to the climate of the Southern Hemisphere) and a small effect of latitude. Overall, adolescent girls had the lowest vitamin D status. The effect of skin pigmentation or sunscreen usage could not be evaluated. The between-person variation was, as usual around the world, great and exceeded by far the seasonal variation. Indeed, with a standard deviation of 9 ng/mL, the range of ± 2SD would be 11 - 57 ng/mL (if a Gaussian distribution would apply). The reasons for this large variation remain unexplained and clarification of this variation might help in identifying the persons most at risk for vitamin D deficiency and the best focus for targeted supplementation. The paper fortunately also included essential quality control samples to evaluate the accuracy of the 25(OH)D assay. Using DEQAS samples with liquid chromatography-tandem mass spectrometry (LC-MS/MS) verified sera samples, the immunoassay used in the study revealed a relatively small bias of about 6.0 %.^2^ Ideally, for such kind of large studies, it would be wise to recalibrate the results using well-described methodologies.^9^

Is the vitamin D status of children very different from the vitamin D status in adults around the world? There are thousands of studies reporting the vitamin D status in different areas of the world but the large majority deals with adults and elderly subjects. A recent overview comparing the situation in different continents concluded that about 7.0 % of the world's population lived with serum 25(OH)D concentrations below 12 ng/mL. This was also the frequency of such severe deficiency in the NHANES study (dealing with samples from subjects older than 12 years), whereas higher percentages were found in European countries (14.0 %) and even much higher percentages in the Middle East or Gulf states, North African countries, Mongolia and Northern China. The lowest rate was found in some equatorial countries such as Ghana and the Seychelles.^10^ There are fewer data dealing with children and adolescents but in general vitamin D status of children is not much different from that of adults living in the same area. Table 1 11, 12, 13, 14, 15, 16, 17, 18, 19, 20, 21, 22, 23, 24, 25, 26, 27, 28, 29, 30, 31, 32, 33, 34, 35, 36, 37, 38, 39, 40, 41, 42, 43, 44, 45, 46, 47 summarizes the data obtained in different countries and demonstrates a wide range of situations around the world. There are different ways to look at the results of this large survey. Evidently, one would prefer that no child should live with vitamin D deficiency. Comparison with other areas around the world can also help to interpret the results. From Table 1 it is obvious that severe vitamin D deficiency (below 30 nmol/L or 12 ng/mL) is rare in Brazil (0.8 %) and therefore no country in the world can really be better than the virtual absence of such a severe form of deficiency. A more modest deficiency was found in 12.5 % of the Brazilian children in the present study by Radonsky et al. Only two countries were better, South Africa and Canada. The Canadian data can probably be explained by the younger age groups reported (0 - 5 years) and the high rate of implementation of vitamin D supplementation in young children in general. Most countries thus perform less well than Brazil and some areas or countries are being confronted by a more than 30.0 – 50.0 % risk of modest deficiency, e.g., Italy (and some other European countries), Lebanon (and probable several North African and Gulf states), Mongolia (and probably upper China and India) and Korea.

**Table 1:** An overview of the major studies on the vitamin D status of children in different areas of the world. The table is not exhaustive but presents a representative overview of the largest studies. NA, not available.

	Geographical area	Study population	Study size (n)	Baseline data	Prevalence,%	
					Cutoff of < 20 ng/mL (50 nmol/L)	Cutoff of < 8 - 12 ng/mL (20 - 30 nmol/L)
Isa et al. (2020)^18^	Bahrain	0 – 16 years	531	2016	78.3 %	–
Radonsky et al. (2024)^8^	Brazil	0 – 18 years	413,976	2014 – 2018	12.5 %	0.8 %
Santos et al. (2012)^19^	Brazil	7 – 18 years	234	2008 – 2011	36.3 %	–
Lourenço et al. (2020)^20^	Brazil	0 – 2 years	468	2012 – 2013	68.2 %	32.9 %
Maguire et al. (2013)^16^	Canada	1 – 5 years	1898	2008 – 2011	6.0 %	–
Yang et al. (2020)^21^	China	0 – 18 years	460,537	2016 - 2018	22.6 %	6.7 %
Li et al. (2022)^22^	China	0 – 18 years	10,262	2012 – 2020	14.0 %	–
Zhang et al. (2020)^23^	China	0 – 6 years	6953	2016	22.1 %	–
Gonzalez-Gross et al. (2012)^11^	Europe	12.5 – 17.5 years	1006	2006 – 2007	42.0 %	15.0 %
Cashman et al. (2016)^24^	Europe	1 – 17 years	10,015	2002 – 2012	44.5 %	6.0 %
Wolters et al. (2022)^25^	Europe	3 – 15 years	2171	2007 – 2014	63.0 %	–
Lehtonen-Veromaa et al. (1999)^26^	Finland	9 – 15 years	191	1997 – 1998	–	13.4 %
Absoud et al. (2011)^27^	Great Britain	4 – 18 years	1102	1997 – 1998	35.1 %	–
Marwaha et al. (2005)^28^	India	10 – 18 years	5137	NA	–	35.7 %
Chowdhury et al. (2019)^29^	India	0 – 3 years	960	2010 - 2012	–	34.5 %
Rabbani et al. (2009)^30^	Iran	7 – 18 years	963	NA	34.9 %	6.7 %
Carson et al. (2015)^31^	Ireland	12 – 15 years	1015	1999 – 2000	66.2 %	16.7 %
Korchia et al. (2013)^32^	Israel	1 – 6 years	247	2009 – 2010	28.3 %	–
Vierucci et al. (2013)^12^	Italy	2 – 21 years	652	2010 – 2012	45.9 %	9.5 %
Lippi et al. (2007)^33^	Italy	0 – 18 years	192	2004 – 2007	–	6.2 %
Marrone et al. (2012)^34^	Italy	0 – 18 years	93	2009 - 2010	54.8 %	–
Mazzoleni et al. (2012)^35^	Italy	1 – 15 years	58	2010 – 2011	50.0 %	12.1 %
Nichols et al. (2015)^36^	Jordan	1 – 5 years	1077	2010	56.5 %	19.8 %
Kim et al. (2014)^13^	Korea	10 – 18 years	2062	2008 – 2009	68.1 %	13.4 %
El-Hajj Fuleihan et al. (2001)^37^	Lebanon	10 – 16 years	169	1999	65.0 %	21.0 %
Flores et al. (2021)^38^	Mexico	1 – 4 years	1209	2018 – 2019	27.3 %	–
Flores et al. (2021)^38^	Mexico	5 – 11 years	3482	2018 – 2019	17.2 %	–
Ganmaa et al. (2020)^39^	Mongolia	6 – 13 years	8851	2015 - 2017	95.6 %	31.8 %
Holten-Andersen et al. (2020)^40^	Norway	0 – 18 years	301	2015 – 2017	22.0 %	1.0 %
Al Shaikh et al. (2016)^41^	Saudi Arabia	6 – 15 years	2110	2013 – 2014	95.3 %	45.4 %
Poopedi et al. (2011)^42^	South Africa	10 years	385	NA	7.0 %	–
Karagüzel et al. (2014)^43^	Türkiye	11 – 18 years	746	2010	93.0 %	–
Das et al. (2006)^44^	United Kingdom	14 – 17 years	51	2003	–	73.0 %
Gordon et al. (2004)^45^	USA	11 – 18 years	307	2001 – 2003	42.0 %	4.6 %
Dong et al. (2010)^14^	USA	14 – 18 years	559	2001 – 2005	28.8 %	5.2 %
Mansbach et al. (2009)^46^	USA	1 – 11 years	4558	2001 – 2006	18.0 %	1.0 %
Gordon et al. (2008)^47^	USA	0 – 2 years	380	2005 - 2007	12.1 %	–

## Summary and conclusions

• Vitamin D deficiency, however defined, is frequent around the world.
• There is no unanimity about which serum 25(OH)D is sufficient for global health. An overview of guidelines concluded that there is great unanimity that serum 25(OH)D concentrations below 12 ng/mL should be avoided at all ages. The UK guidelines prudently concluded that there are no convincing randomized controlled trials that higher levels than 12 ng/mL convey additional benefits. However, most other guidelines recommended a minimal threshold of 20 ng/mL to avoid secondary hyperparathyroidism and its long-term consequences. The Endocrine Society and some other scientific organizations, including the Brazilian Endocrine Society, preferred a serum concentration of at least 30 ng/mL especially because of observational studies linking such “insufficiency“ (serum 25(OH)D between 20 - 30 ng/mL) with major health consequences such as cancer, metabolic and cardiovascular diseases. Finally, a few experts believe serum 25(OH)D should be about or above 40 ng/mL as this level is found in a few tribes living as our early ancestors probably did.
• The vitamin D status of Brazilian children is certainly much better than in most moderate climates. Severe vitamin D deficiency (< 12 ng/ml) is rare at overall levels below 1.0 %, compared with 15.0 % in Europe,^11^^,^^12^ and 13.0 % in Korea.^13^ Similarly, low frequency of severe vitamin D deficiency was also found in Southern USA,^14^ China,^15^ or Canada.^16^ The rickets consensus^1^ and most guidelines consider such low serum 25(OH)D to be a risk factor for rickets. Fortunately, not all children with such low serum 25(OH)D develop rickets and it is unclear what other factors, other than very low calcium intake, are involved in causing this severe disease. Rickets is unfortunately still far too frequent in some regions of the world. The WHO should take up a leading role in formulating strategies and implementation policies to eradicate nutritional rickets.
• Vitamin D deficiency can relatively easily be corrected. A population-based evaluation of serum 25(OH)D is an excellent strategy to determine to what degree such deficiencies exist, and which strategies could be used to correct such deficiencies. The present study used convenient sampling using existing results of measurements of serum 25(OH)D. Due to its very large number of children from different areas of Brazil, it probably represents a good proxy for the real situation.
• Modest vitamin D deficiency is highly prevalent in many countries. Brazil is rather well off in comparison with other countries, but of course, 12.5 % of the population is modestly deficient, and higher rates in some subjects in wintertime, require specific attention. For some countries where the majority of the population has such a deficiency, food fortification is probably the best strategy as shown by the Finnish example. For Brazil, a more dedicated approach may be more appropriate. One such option could be to recommend a daily or weekly dose of vitamin D (about 600–1000 IU/d) for subjects most at risk such as adolescent girls during wintertime.
• Whether achieving levels higher than 20 ng/mL would convey additional benefits is yet unclear. The recent large mega-trials in the US, Australia and New Zealand concluded that vitamin D supplementation of adults, considered vitamin D replete on the basis of serum 25(OH)D well above 25 ng/mL, did not generate beneficial effects on cardiovascular or metabolic risks and also did not decrease cancer incidence. Similar studies in children are not available. There are, however, more solid data linking a good vitamin D status with beneficial immune effects, but the available data do not allow us to define which 25(OH)D levels are needed for such effects.
• Correction of vitamin D deficiency should always be complemented by assuring adequate calcium intake. Overall, there is room for improvement in the calcium intake of children in Brazil as shown by a large population-based study.^17^

## Conflicts of interest

The authors declare no conflicts of interest.
